# Malaria vaccine roller coaster

**DOI:** 10.1038/s41564-021-00982-0

**Published:** 2021-11-01

**Authors:** Irene N. Nkumama, Faith H. A. Osier

**Affiliations:** 1 Centre of Infectious Diseases, Parasitology Unit, Heidelberg University Hospital, Heidelberg, Germany; 2 IAVI Human Immunology Laboratory, Imperial College London, London, UK

## Abstract

A promising vaccine fails to provide durable protection against infection and clinical malaria in infants, a key malaria vaccine target population, in a phase 2b clinical trial. The need for a highly effective vaccine against malaria remains as urgent as ever.

Malaria vaccine development has been characterised by excitement and disappointment in equal measure. Candidates that have shown promise in pre-clinical animal models and phase 1/2 clinical trials in volunteers who are malaria naive typically do not make the grade when tested in malaria-endemic populations. Reporting in *Nature Medicine*, Oneko et al.^1^ document the finding that the PfSPZ (Sanaria) malaria vaccine does not protect Kenyan infants against malaria infection in a phase 2 randomised controlled trial. A total of 336 infants aged 5–12 months received 4.5 × 10^5^, 9.0 × 10^5^ or 1.8 × 10^6^ sporozoites by direct venous injection, with normal saline as a control.

There are over 200 million clinical cases of malaria each year and approximately 400,000 deaths. *Plasmodium falciparum* parasites are responsible for the most severe forms of malaria and have a complex life cycle. Infected *Anopheline* mosquitoes inject sporozoites into the skin, which then traffic to the liver, invade hepatocytes and develop to release tens of thousands of merozoites that subsequently infect erythrocytes. Merozoites mature and multiply asexually, causing repeated cycles of erythrocyte rupture, egress and reinvasion, which are associated with the clinical symptoms of malaria. The parasite life cycle is completed when a proportion of merozoites develop into sexual gametocytes that are taken up by a mosquito during a blood meal, form zygotes within its midgut and develop into sporozoites. Malaria vaccines target single or multiple stages of this complex life cycle, may include single or multiple antigens or are based on attenuated versions of the whole organism.

PfSPZ is a live radiation-attenuated whole organism vaccine (metabolically active but non-replicating) that aims to prevent malaria infection by inducing immune responses targeting the sporozoite stage. It builds on a series of early studies demonstrating that attenuated sporozoites delivered through the bites of irradiated infected mosquitoes could induce sterile protection by preventing the establishment of infection^2^. Vaccines ultimately need to be delivered in a format that is easy to mass produce, distribute, store and deliver, leading investigators to develop injectable versions of attenuated sporozoites that met regulatory requirements. Interestingly, neither subcutaneous nor intradermally injected radiation-attenuated sporozoites conferred protection and both induced only low-level immune responses^3^. However, intravenous administration (IV) of PfSPZ in non-human primates elicited strong PfSPZ-specific T-cell responses and had >80% protective efficacy in mice^3^. These encouraging findings set the scene for human trials.

Excitement about the PfSPZ vaccine increased when IV vaccination with PfSPZ led to 100% sterile protection in a cohort of 40 adults who were malaria naive^4^, but the feasibility of such an approach in rural Africa and similar resource-constrained settings remained a concern. Furthermore, in subsequent studies, vaccine efficacy was lower in adults who were exposed to malaria as compared to those who were malaria naive^5^, reminiscent of previous unsuccessful malaria vaccines. This led to the hypothesis that previous exposure to *P*. *falciparum* interfered with the development of robust immune responses following vaccination with PfSPZ and formed the basis for a comparative study in infants, children and adults in Tanzania^6^. Somewhat in keeping with the hypothesis, infants vaccinated with the highest dose of PfSPZ had the highest levels of antibodies as compared to children and adults. Of note, although animal models and other human studies suggested that T-cell responses were important for protection^3^, no PfSPZ-specific T-cell responses were detected in the Tanzanian infants. In the current study, there was neither prevention of infection at six months post-vaccination, the primary end point, nor induction of PfSPZ-specific T-cell responses^1^ (Fig. 1). Moreover, although a dose-dependent increase in antibodies was observed in all age groups, its contribution to the modest vaccine efficacy observed at three months (exploratory end point) is less clear.

As investigators draw the curtain on the administration of PfSPZ to infants, the use of this vaccine remains an option for other age categories, subject to the outcomes of ongoing trials, and illustrates important, unanswered questions about malaria vaccines. The most important is the lack of a universally accepted immune correlate of protection. Although Oneko et al. predicated the trial partly on the finding that PfSPZ-induced antibodies were highest in infants as compared to adults, they propose that the lack of priming of γδ T cells in these infants is one explanation for the negative outcomes. Moreover, although protection against clinical disease was observed at three months post-vaccination, both the non-protected high-dose group and protected low-dose group had similar antibody levels, suggesting that additional factors are important.

The immunological mechanisms that underpin natural and vaccine-induced immunity are also unclear. Recently, studies have revealed a role for Fc-mediated antibody effector functions in protection against *P*. *falciparum* infection^7^. The impact of prior exposure to malaria on vaccine-induced responses has not yet been ascertained. Pre-existing naturally acquired antibodies may mask or compete for important epitopes or may hinder the activation of memory B cells. Plus, immunomodulation by regulatory T cells has been shown to have a role in immune responses to malaria. Therefore, conducting early (phase 1/2) clinical trials in malaria-endemic countries may be instructive. Another potential contributor to the low efficacy of vaccines in endemic areas is parasite diversity^8^. Although previous studies using PfSPZ showed that a higher inoculum was necessary for protection following a heterologous (different parasite strain) challenge in a human trial, it is unclear whether this will apply to the level of diversity experienced under natural conditions. Finally, strategies to induce durable protection and ideally life-long protection are needed. Regular boosting could be considered but would need to be weighed against implementation challenges in real-world conditions. Ideally, the timing of boosters would fit into the schedule of the well-established expanded programme of immunization that protects against a growing number of childhood infections.

Alternative methods for *P*. *falciparum* attenuation are also in development. Genetically attenuated parasites incorporate mutations that lead to the arrest of parasite development in the liver in humans. Fully infectious sporozoites are also being administered concurrently with prophylactic anti-malarial drugs (for example, chloroquine and mefloquine) to arrest parasite growth in the first blood-stage cycle. Both strategies could enable the expression of more parasite antigens and may result in the induction of more durable immune responses. The most advanced of the subunit vaccines is RTS,S, which is based on the circumsporozoite protein (CSP) and is currently undergoing post-licensure implementation in three African countries. A similar vaccine, R21, has a high density of CSP presented on virus-like particles and a recently reported vaccine efficacy of >75%, the highest for a malaria vaccine to date^9^, in Burkinabe infants aged 5–17 months who had been exposed to malaria. However, this study was conducted in a region of highly seasonal malaria transmission, and antibody levels waned during the months post-vaccination, with practically no malaria transmission, which means that the outcomes may not be broadly applicable in other populations and locations. Validation in independent studies and varied transmission settings will provide further clarity. New vaccine platforms like mRNA and monoclonal antibodies are currently being explored, with the latter showing promise in an early human challenge study^10^.

The fact that humans can acquire protective immunity to malaria provides strong impetus for vaccine development. The hope is that coupling robust immune correlates with technological advances in vaccinology will be successful because the need for malaria vaccines remains as pressing now as it was over a century ago.

## Figures and Tables

**Fig. 1:**
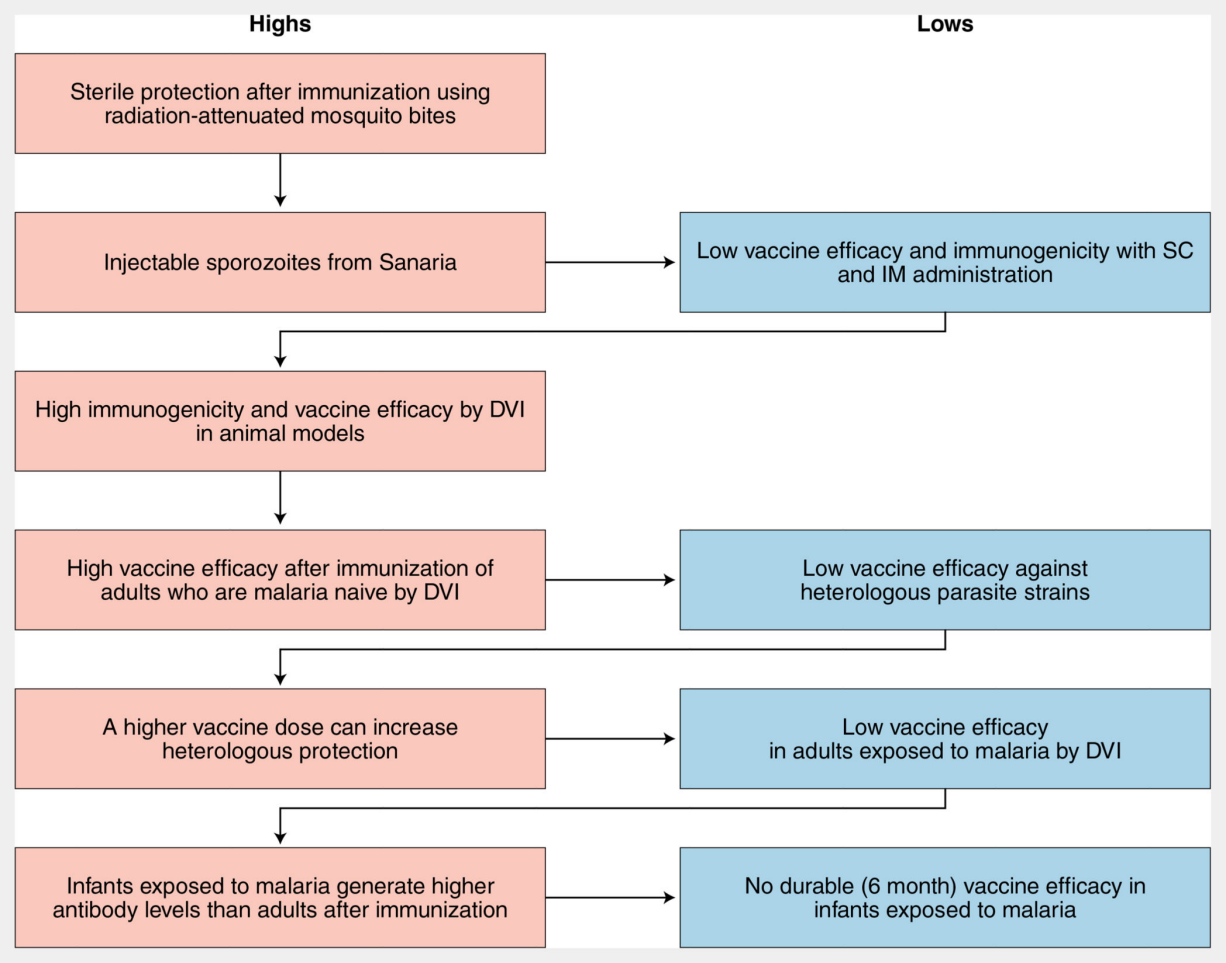
The highs and lows of PfSPZ malaria vaccine development. A flow chart of notable exciting and disappointing moments in the course of developing the PfSPZ vaccine. DVI, direct intravenous injection; IM, intramuscular; SC, subcutaneous.
